# Editorial: Regulatory action of calcium channels in pain pathway

**DOI:** 10.3389/fncel.2022.928457

**Published:** 2022-08-15

**Authors:** Senthilkumar Rajagopal, Divya P. Kumar, Albert Baskar Arul

**Affiliations:** ^1^Department of Biotechnology, School of Applied Sciences, REVA University, Bangalore, KA, India; ^2^Department of Biochemistry, JSS Medical College, CEMR, JSS Academy of Higher Education and Research, Mysore, KA, India; ^3^Department of Chemistry, RASR Laboratory, Vanderbilt University, Nashville, TN, United States

**Keywords:** voltage-gated calcium channels, protein kinas C (PKC), calcium influx, bradykinin, pregabalin

Pain is a distressing feeling often caused by intense or damaging stimuli. In order to address this challenge and improve the lives of people affected by pain conditions, better understanding of the mechanisms of pain and improved treatments are needed. Acute pain can be mild and momentary. Pain is classified into three categories: nociceptive, neuropathic, and inflammatory (Bourinet et al., 2014). Nociceptive pain results from activity in neural pathways and is the most common form of chronic pain. It encompasses arthritis and most forms of spinal pain. Neuropathic pain is caused by damage or disease affecting the somatosensory nervous system and is typically associated with sensory abnormalities such as numbness and alloydynia. Approximately 15–25 % of chronic pain is neuropathic and includes diabetic neuropathy, postherpetic neuralgia, and radiculopathy. Nociplastic pain is pain arising from the abnormal processing of the pain signals without any clear association with damage, injury, or disease pathology (Cervero, 2000; Finnerup et al., 2013). Figure 1 is a schematic diagram that depicts various pain stimulates and major calcium channels involved in pain pathways.

**Figure 1:**
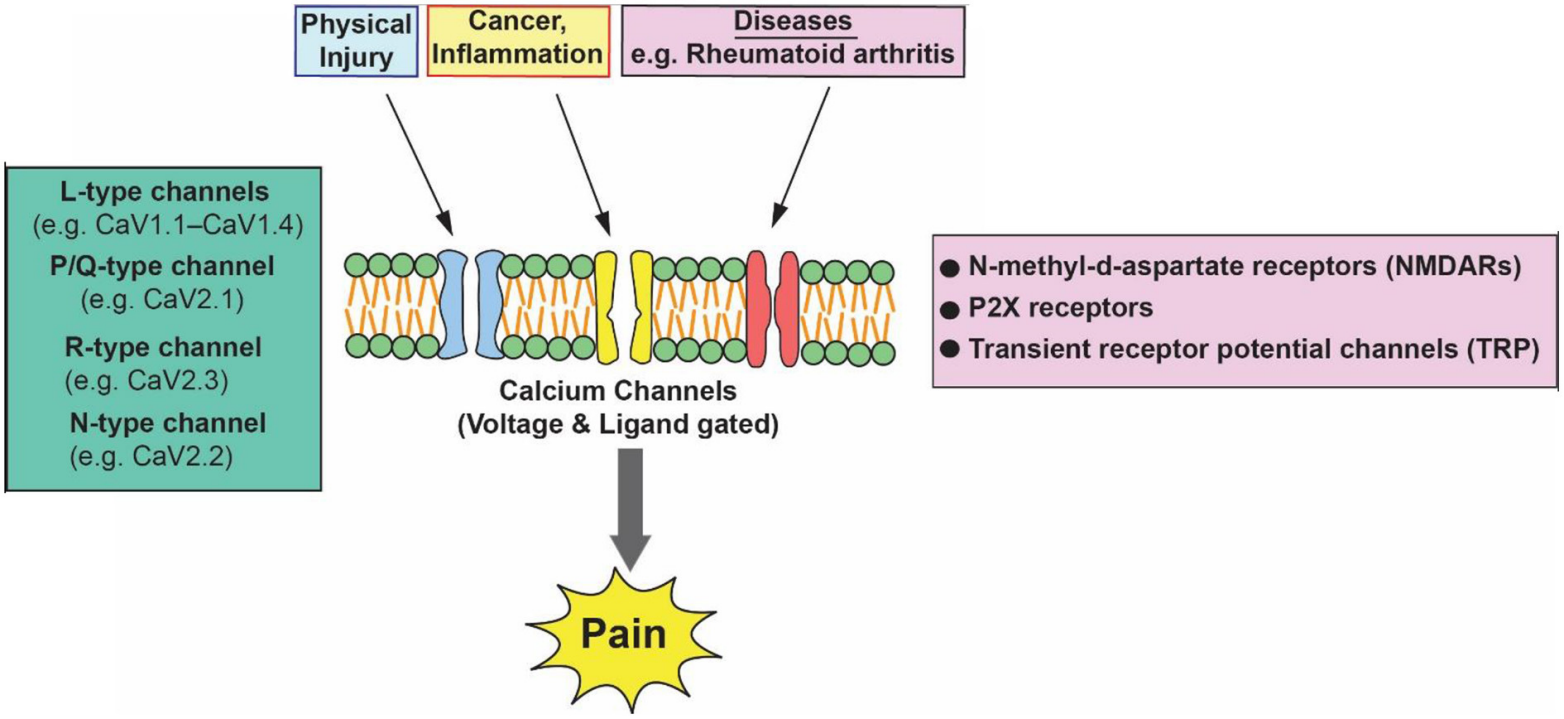
Regulatory action of voltage-gated calcium channels in pain pathways.

Pain mediators such as bradykinin, serotonin, substance P, and prostaglandin E2 increase Ca^2+^-influx through CaV leading to a significant increase in intracellular calcium ([Ca^2+^]i). The increased ([Ca^2+^]i) signal can contribute to an increase in neural activity which is relayed to the central nervous system and leads to increased pain perception (Cervero, 2000; Senthilkumar et al., 2010). Previous studies have shown that chronic changes in ion channel expression and function are thought to partially contribute to chronic pain. These ion channels mediate cell signaling, as well as regulate membrane potential and excitability functions. These functions include the release of neurotransmitters, the activation of calcium-dependent enzymes, and calcium-dependent changes in plasticity and gene transcription (Patel et al., 2017; Senthilkumar and Murugavel, 2017).

This Research Topic entitled “*Regulatory Action of Calcium Channels in Pain Pathway*” features review articles, a perspective article, and an original article that sheds light on the role of calcium channels in the modulation of pain signals in health and disease.

Joksimovic et al., provide experimental evidence of how post-translational modification, such as glycosylation, adversely affects the biophysical property of CaV3.2 channels. This leads to the development of hyperalgesia in type 1 peripheral diabetic nephropathy. Furthermore, it has been demonstrated that deglycosylation by the administration of glycosylation inhibitors, such as neuraminidase and PNGase-F, potentially ameliorated the pain in STZ (streptozotocin) for treated diabetic mice and rats.

Cho and Huh review literature and discuss how calcium channels in astrocytes play a critical role in chronic pain development. The role of various calcium sources and their contribution to different types of reactive astrocytes, which may have different effects on chronic pain, are well described. This indicates that astrocytic calcium may offer better targets for pain control.

In their perspective article, Muller and Reggio analyze the putative cannabidiol (CBD) binding site in the transient receptor potential (TRP) ion channels. CBD, a cannabinoid ligand, is known to modulate different types of TRP channels such as TRPV1, TRPV2, TRPV3, TRPV4, TRPA1, and TRPM8. These channels are implicated in inflammation and chronic pain. The study clearly describes the differences that exist in the putative CBD binding sites and how this can be exploited in targeting the endocannabinoid system for the modulation of pain.

Alles et al. discuss the role of pregabalin, a gabapentinoid that exhibits a high affinity to voltage-gated calcium channels, and its therapeutic role in the treatment of chronic pain. The review article discusses the alpha 2 delta (α2δ) subunits of calcium channels in pathophysiology and how pregabalin selectively binding to α2δ1 and α2δ2 subunits performs its action in the alleviation of acute and chronic pain.

Studies by Behrendt et al., have elucidated the signaling pathways involved in bradykinin (BK)-induced Transient Receptor Potential Channel Melastatin 3 (TRPM3) sensitization during inflammation. DAG kinase, elevated cytosolic calcium, and vesicular exocytosis are all involved in the sensitization process, signifying how elevated TRPM3 expression could lead to altered heat sensitivity during inflammation. The research work by Zhi et al. provides the molecular basis for somatostatin-positive (SOM+) neurons in transmitting mechanical pain. Furthermore, the functional role of the T-type calcium channel CaV 3.2 has also been revealed in tactile and pain processing at the level of the spinal cord.

Taken as a whole, this Research Topic aims to provide an overview of the different calcium-permeable ion channels involved in pain processing pathways.

## Author contributions

SR, DK, and AA: substantial contributions to the conception, design of the work, drafting the work or revising it critically for important intellectual content, provide approval for publication of the content, and agree to be accountable for all aspects of the work in ensuring that questions related to the accuracy or integrity of any part of the work are appropriately investigated and resolved.

## Conflict of interest

The authors declare that the research was conducted in the absence of any commercial or financial relationships that could be construed as a potential conflict of interest.

## Publisher's note

All claims expressed in this article are solely those of the authors and do not necessarily represent those of their affiliated organizations, or those of the publisher, the editors and the reviewers. Any product that may be evaluated in this article, or claim that may be made by its manufacturer, is not guaranteed or endorsed by the publisher.

## References

[B1] Bourinet E. Altier C. Hildebrand M. E. Trang T. Salter M. W. Zamponi G. W.. (2014). Calcium-permeable ion channels in pain signaling. Physiol. Rev. 94, 81–140. 10.1152/physrev.00023.2013 24382884

[B2] Cervero F. (2000). Visceral pain-central sensitization. Gut 47, 56–57. 10.1136/gut.47.suppl_4.iv56 11076916 PMC1766826

[B3] Finnerup N. B. Scholz J. Attal N. Baron R. Haanpää M. Hansson P.. (2013). Neuropathic pain needs systematic classification. Eur. J. Pain 17, 953–956. 10.1002/j.1532-2149.2012.00282.x 23339030

[B4] Patel R. Montagut Bordas C. Dickenson A. H. (2017). Calcium channel modulation as a target in chronic pain control. Br. J. Pharamcol. 175, 2173–2184. 10.1111/bph.13789 28320042 PMC5980588

[B5] Senthilkumar R. Fang H. Lynch I. I. I. C. Kamatchi G. L. (2010). Formalin -induced -short and long-term modulation of Cav currents expressed in Xenopus oocytes: an in vitro cellular model for formalin-induced pain. Basic Clin. Pharmacol. Toxicol. 106, 338–347. 10.1111/j.1742-7843.2009.00496.x 20030632

[B6] Senthilkumar R. Murugavel P. (2017). Calcium Signaling: Physiology to diseases. Chapter 5: Voltage dependent calcium channels: From Physiology to diseases. Singapore: Springer Nature publications, 61–69. 10.1007/978-981-10-5160-9_5

